# Clinical Analysis for Long-Term Sporadic Bovine Viral Diarrhea Transmitted by Calves with an Acute Infection of Bovine Viral Diarrhea Virus 2

**DOI:** 10.3390/v13040621

**Published:** 2021-04-04

**Authors:** Yusuke Goto, Gakuji Yaegashi, Kazuhiro Fukunari, Tohru Suzuki

**Affiliations:** 1 Central Iwate Prefectural Livestock Health and Hygiene Center, Takizawa, Iwate 020-0605, Japan; y.gotou@pref.iwate.jp (Y.G.); g-yaegashi@pref.iwate.jp (G.Y.); k-fukunari@pref.iwate.jp (K.F.); 2 Division of Pathology and Pathophysiology, Hokkaido Research Station, National Institute of Animal Health, NARO, Sapporo, Hokkaido 062-0045, Japan

**Keywords:** bovine viral diarrhea virus, acutely infection, calves, virus transmission

## Abstract

Bovine viral diarrhea virus (BVDV) is a viral pathogen associated with serious problems in the cattle industry. Cattle persistently infected (PI) with BVDV are mild or asymptomatic; however, they become a source of BVDV transmission to other cattle. Hence, it is important to rapidly identify and remove the PI animals from cattle herds. Whereas cattle acutely infected (AI) with BVDV have various symptoms, yet they generally recover within 3 weeks. However, there is a paucity of information concerning clinical characteristics of AI cattle. Further accumulation of information would be required to accurately diagnose AI cattle with BVDV. Here, we attempted to obtain valuable information via various analyses using a case report of BVD outbreak that occurred for approximately four months in Iwate Prefecture in 2017. Using eight calves and multiple tests (real-time RT-PCR, virus isolation, enzyme-linked immunosorbent assay, and virus neutralization assay) over 6 weeks, we diagnosed the continuous BVD outbreak as an acute infection and not a persistent one. Additionally, we revealed that the sporadic case was caused by low pathogenic BVDV2 via BVDV genotyping and phylogenetic analysis. The data suggest that BVDV2 AI animals might also be a source of transmission to susceptible calves; hence, it might persist for a long period owing to multiple AI animals. These findings provide useful information to diagnose AI and PI cattle with BVDV in the field.

## 1. Introduction

Bovine viral diarrhea virus (BVDV) is one of the most important viral pathogens among cattle disorders with a worldwide economic impact [1,2,3,4]. BVDV is a positive single-stranded RNA virus, which belongs to the genus *Pestivirus* of the family *Flaviviridae*, which includes border disease virus and classical swine fever virus [5].

BVDV is currently classified into three different genotypes (BVDV1–3) based on their antigenic characteristics [6,7,8]. A previous study reported that in recent years, BVDV1 and BVDV2 had a 71% and 29% occurrence frequency of the genotypes within Japan, respectively [9]. Although there are some previous reports on the existence of highly pathogenic BVDV2, such as the 890 strain isolated from United States and the 1373 strain isolated from Canada, there are no reports of such strains in Japan [9,10].

BVDV infection is associated with similar clinical symptoms despite having three different genotypes [10,11,12]. Especially, BVDV infections of pregnant cows during approximately 45–125 days of gestation is related to births of persistently infected (PI) calves [13,14]. The PI calves exhibit mild or no symptoms; however, they continuously excrete viruses into environment throughout their lifetime, and are thus problem sources of BVDV transmission within a herd [4,15,16,17].

Cattle acutely infected (AI) with BVDV experience various symptoms such as slight fever, leukopenia, respiratory disorders, diarrhea, and abortion [13,18,19]. In addition, AI animals seronegative to BVDV showed a short-term (approximately 4–10 days) of viremia and virus shedding in nasal and various other discharges [20,21]. In several cases, AI animals turned from serologically negative into positive within 2 weeks after BVDV infection; however, they recovered 3 weeks after the onset of BVDV infection [22,23,24,25]. In a previous study, AI calves were known to not be a source of BVDV transmission because of mild or no symptoms and low excretion of BVDV, compared with PI calves [10,24,26,27,28,29]. In contrast, other studies showed that BVDV antibodies turned positive in cattle herds without PI calves; however, the details remain unclear [20,30]. Therefore, it is crucial to accumulate clinical information for BVDV acute infection, such as disease process, virus excretion, and virus transmission via various analyses of AI cattle [24].

We found a case report of AI by BVDV2 that persisted for four months in a dairy cattle farm without PI calves. This is one of study to demonstrate clinical characteristics of this disease via a series of analyses for the occurrence of continuous AI by low pathogenic BVDV2 in Japan.

## 2. Materials and Methods

### 2.1. The Bovine Farm

A total of 147 cows, 33 heifers, and 24 calves were maintained in a dairy farm which consisted of two cattle houses; one was a dairy barn containing a delivery room and the other was a calf barn. Both houses were approximately 5 km apart, and had different workers between them. 

**Figure 1:**
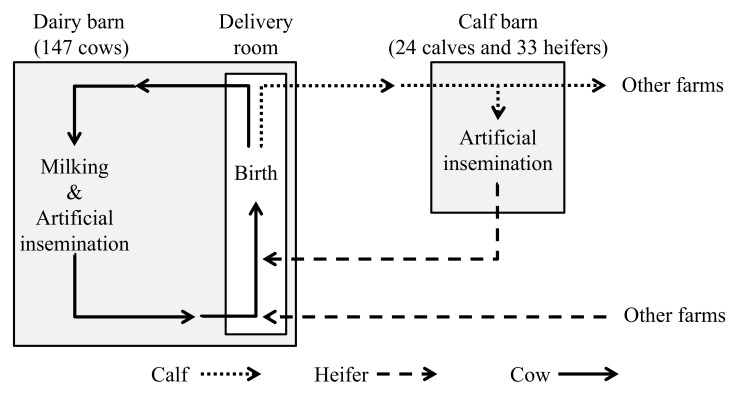
A flow chart for the movements of calves, heifers, and cows maintained in this farm. Calves born at the delivery room were transferred to the calf barn on the day of their birth. Both houses were approximately 5 km apart and the workers were different in both houses. Most heifers were kept at the calf barn until one month before delivery, and directly returned to the delivery room. Some heifers were transferred to other farms when they were calves, and kept there until one month before delivery. Delivered cows were kept at the dairy barn, in which milking and artificial insemination were performed.

The flow of cattle movement is shown in Figure 1. Newborn calves were carried to the calf barn on the day of their birth. The newborn calves were usually fed pooled colostrum which had been collected and stocked from the delivered cows. In addition, most heifers were artificially inseminated at the calf barn, and subsequently moved to the delivery room approximately one month before delivery. Some heifers were reared and underwent artificial insemination on other farms, and returned to the delivery room before delivery. Of the 147 cows maintained in this farm, 53 cows had been transferred and reared into other farms before. An inactivated vaccine that combined BVDV1 with BVDV2 was not used in this farm.

### 2.2. Occurrence of Bovine Enteric and Respiratory Disorders among Cattle Herd Maintained at Calf Barn

In March 2017, two calves (nos. 569 and 664) presented with diarrhea. We defined the day of sample collection from both calves as Day 0. Thereafter, two calves (nos. 663 and 835) exhibited respiratory symptoms on Day 13 and Day 38, respectively. Moreover, three calves (nos. 772, 597, and 711) and one calf (no. 761) exhibited respiratory symptoms on Day 42 and bloody stool and respiratory symptoms on Day 76, respectively. Three of eight calves (no. 835, and nos. 711 and 761) died on Day 47 and 76, respectively. In addition, one calf (no. 664) was euthanized because of poor growth on Day 102. Finally, we observed the symptoms demonstrated by these animals until Day 120. Other than the eight calves, three calves exhibited respiratory symptoms or diarrhea among other cattle maintained at calf barn. 

### 2.3. Diagnosis of Eight Calves Based on a Series of Analyses

#### 2.3.1. Sampling

**Figure 2:**
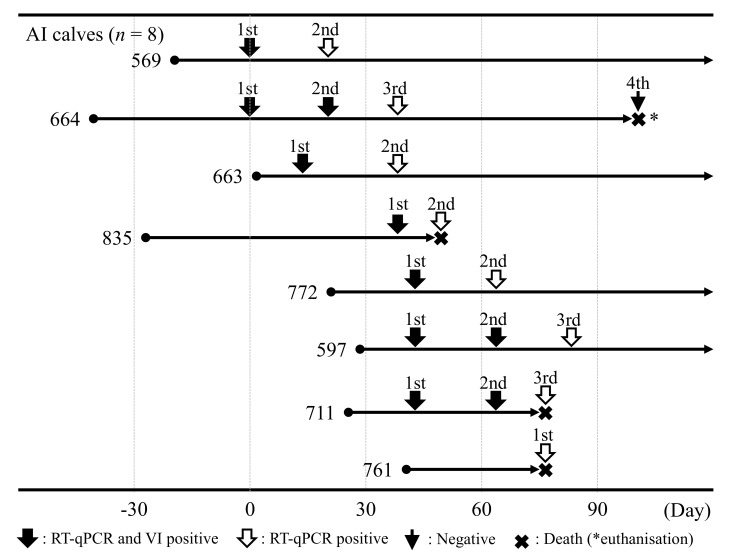
Sampling scheme of eight calves with clinical symptoms. The day samples were collected from the two calves (nos. 569 and 664) with diarrhea was defined as Day 0. The numbers represent the individual number of the eight calves. Filled bold-arrows represent positive results for BVDV via RT-qPCR and virus isolation. White bold-arrows represent positive results for BVDV via only RT-qPCR. Thin-arrow represents negative results for BVDV via RT-qPCR and virus isolation. * euthanization.

A sampling scheme for eight calves is shown in Figure 2. Sera and white blood cells (WBCs) were collected one to three times from these calves at 3-week intervals from Day 0 to Day 84. Several tissues (superficial cervical lymph node, subiliac lymph node, kidney, spleen, liver, lung, heart, and brain) were collected from three dead calves and one euthanized calf. These tissues were used to make 20% suspensions by homogenization with Eagle’s minimum essential medium (EMEM) (Nissui Pharmaceutical, Tokyo, Japan). All samples were collected as a part of routine diagnostic procedures, hence, permission concerning animal ethics was not required.

#### 2.3.2. RNA Extraction and Real-Time RT-PCR for the Detection of Bovine Viral Diarrhea Virus

Viral RNA was extracted from sera, WBCs, and 20% tissue suspensions using the QIAamp Viral RNA Mini Kit according to the manufacturer’s instructions (Qiagen, Hilden, Germany). 

The VetMAX-Gold BVDV PI Detection Kit (Thermo Fisher Scientific, Waltham, MA, USA) was used for real-time RT-PCR (RT-qPCR). The reaction volume of 25 μL was composed of 12.5 μL 2× RT-PCR buffer, 1 μL 25× BVDV primers and probe mixture, 1 μL 25× RT-PCR enzyme mix, 2.5 μL nuclease free water, and 8 μL sample RNA. RT-qPCR was performed using Applied Biosystems 7500 Real-Time PCR systems (Thermo Fisher Scientific) under the following conditions: 45 °C for 10 min and 95 °C for 10 min, followed by 40 cycles of 95 °C for 15 s and 60 °C for 45 s. The results obtained from RT-qPCR were analyzed using ABI 7500 software version 2.0. Based on the manufacturer’s instructions, samples with Ct value <38 were positive for BVDV.

#### 2.3.3. Genotyping of Bovine Viral Diarrhea Virus by Multiplex Real-Time RT-PCR

TaqMan Fast Virus 1-Step Master Mix (Thermo Fisher Scientific) was used for BVDV genotyping by multiplex real-time RT-PCR (multiplex RT-qPCR). Primers and probes were designed with reference to the previous report as follows: a pair of primers (Forward: 5′-GATGCCATGTGGACGAGGGC-3′, Reverse: 5′-CATGTGCCATGTACAGCAGAG-3′) and three modified genotype-specific probes (BVDV1: 5′-FAM-CAATACAGTGGGCCTCTGCAGCA-QSY-3′, BVDV2: 5′-VIC-GTGGCGTTATGGACACAGCCTG-QSY-3′, BVDV3: 5′-ABY-ATCAGGCTGTACTCCCAAAG-QSY-3′) [31]. The reaction volume of 20 μL was composed of 5 μL TaqMan Fast Virus 1-Step Master Mix, 1 μL 20× BVDV1–3 primer and probe mixture, 12 μL nuclease free water, and 2 μL sample of RNA. Multiplex RT-qPCR was performed using Applied Biosystems 7500 Real-Time PCR systems under the following conditions: 50 °C for 5 min and 95 °C for 20 s, followed by 40 cycles of 95 °C for 3 s and 60 °C for 34 s. The results obtained herein were analyzed using ABI 7500 software version 2.0. 

#### 2.3.4. Immunoperoxidase Monolayer Assay for Virus Isolation

Virus isolation from sera, WBCs, and 20% tissue suspensions was performed according to the immunoperoxidase monolayer assay reported previously [32]. Bovine fetal muscular (BFM: originally produced in our laboratory) cells were maintained under following conditions: EMEM supplemented with 3 mg/mL tryptose phosphate broth (Becton Dickinson, San Jose, CA, USA), 0.292 mg/mL L-glutamine (FUJIFILM Wako Pure Chemical Corporation, Osaka, Japan), 1.125 mg/mL sodium hydrogen carbonate (FUJIFILM Wako Pure Chemical Corporation), and 5% BVDV antigen- and antibody-free bovine serum (Japan Bio Serum, Hiroshima, Japan).

Serial twofold dilutions of sera, WBCs, and 20% tissue suspensions were placed into four wells of 96-well plates with 0.1 mL of each dilution. Thereafter, BFM cells (approximately 1.5 × 10^4^ cells) were added into all wells. Plates were incubated at 37 °C in 5% CO_2_ for 4 days, air-dried after medium removal, and fixed at 80 °C for 1 h. Fixed cells were reacted with 50 μL mouse IgG anti-pestivirus monoclonal antibody, JCU/BVD/CF10 (TropBio Pty Ltd., Queensland, Australia) diluted to 1:500 with phosphate-buffered saline (PBS) containing 1% bovine serum albumin (BSA) (FUJIFILM Wako Pure Chemical Corporation) at 37 °C for 30 min. Thereafter, cells were washed twice in PBS containing 0.1% Tween 20 (PBST), and reacted with 50 μL horseradish peroxidase conjugated goat anti-mouse IgG (Bio-Rad Laboratories, Inc. Hercules, CA, USA) diluted to 1:1000 with PBS containing 1% BSA at 37 °C for 30 min. The cells were washed twice in PBST yet again, and reacted with 100 μL solution containing 5% 3-amino-9-ethylcarbazole (AEC) solution (20 mg AEC dissolved in 2.5 mL of dimethyl sulfoxide) and 0.015% H_2_O_2_ in 50 mm acetate buffer (pH 5.0), and incubated in the dark at 37 °C for 30 min. A positive reaction is characterized by the appearance of reddish-brown staining in cytoplasm.

#### 2.3.5. Antigen Detective Enzyme-Linked Immunosorbent Assay (AgELISA)

Serum samples from eight calves were tested via antigen detective enzyme-linked immunosorbent assay (AgELISA) for BVDV using the IDEXX BVDV Ag/Serum Plus Test according to the manufacturer’s instructions (IDEXX Laboratories, Bern, Switzerland). The corrected OD value of the sample was calculated using an absorbance 450 nm obtained from test samples (S) and subtracting the absorbance of the negative control (N). Finally, the calculated OD value was the S-N value. Samples with S-N values ≤0.300 were considered negative for the BVDV antigen. Samples with S-N values >0.300 were considered positive.

#### 2.3.6. Virus Neutralization Test

The virus neutralization test was performed according to a method reported previously [33]. Each 50 μL of serum were serially diluted twofold with 50 μL EMEM supplemented with 3 mg/mL tryptose phosphate broth (Becton Dickinson), 0.292 mg/mL L-glutamine (FUJIFILM Wako Pure Chemical Corporation), 1.125 mg/mL sodium hydrogen carbonate (FUJIFILM Wako Pure Chemical Corporation) on 96-well plates. An equal volume of two representative BVDV strains, Nose (BVDV1) and KZ91-CP (BVDV2), containing 200 TCID_50_ was added to each well, and incubated at 37 °C for 1 h. Thereafter, 100 μL of BFM cells (approximately 1.5 × 10^4^ cells) were added into all wells, and incubated at 37 °C in 5% CO_2_ for 5 days. Appearance of the cytopathogenic effect (CPE) was observed using a microscope (Olympus Corporation, Tokyo, Japan). The virus neutralization titer for each serum was expressed as the reciprocal of the highest dilution that inhibited CPE.

#### 2.3.7. Phylogenetic Analysis for 5′-Untranslated Regions from Bovine Viral Diarrhea Virus Strains

The partial genomes of 5′-UTR (untranslated region) were amplified using the SuperScript III One-step RT-PCR System with Platinum Taq DNA Polymerase (Thermo Fisher Scientific) and primers, 324 (Forward: 5′-ATGCCCWTAGTAGGACTAGCA-3′) and 326 (Reverse: 5′-TCAACTCCATGTGCCATGTAC-3′) that were previously reported [34]. PCR products were determined their nucleotide sequences using the BigDye Terminator v3.1 Cycles Sequencing Kit on an automated ABI Prism 3130 XL Genetic Analyzer (Thermo Fisher Scientific, Carlsbad, CA, USA). Each genomic sequence from the eight BVDVs determined herein was submitted to the DNA Data Bank of Japan; the sequences are retrievable from GenBank (accession numbers: LC600219-LC600226). Phylogenetic analysis using the data added to genomic sequences of other BVDV strains available in GenBank into genomic sequences from the eight BVDV strains was determined using the maximum-likelihood method and 1000 bootstrap replicates on MEGA7 software [35,36]. Nucleotide sequences of other BVDV strains used in this study were as follows: BVDV1 strains, Nose (AB019670) and NADL (AF039181), BVDV2 strains, 1373 (AF145967), 890 (U18059), and KZ91-CP (AB003619). Moreover, this analysis also included four BVDV2 strains from PI animals from Iwate Prefecture between 2012 and 2018 (GenBank accession numbers: LC600227-LC600230).

### 2.4. BVDV Detection of 16 Calves and 33 Heifers Maintained at Calf Barn, 147 Cows Maintained at Dairy Barn, 5 Calves Transferred to Other Farms, and 20 Newborn Calves

#### 2.4.1. Sampling

On Day 42, sera were collected from 16 calves (<1 to 6 months old) and 33 heifers (7 to 22 months old) that were maintained at calf barn, excluding the above eight calves. On Day 95, sera were collected from 147 cows (21 to 107 months old) maintained at dairy barn. In addition, sera were collected from five calves (15 to 83 days old on day of sample collection) before transferred to other farms between Day 0 and Day 41. Moreover, sera were collected from 20 calves (2 to 34 days old on day of sample collection) newly born in this farm between Day 43 and Day 120. All samples were collected as a part of routine diagnostic procedures, hence, permission concerning animal ethics was not required. 

#### 2.4.2. RNA Extraction and Real-Time RT-PCR for the Detection of Bovine Viral Diarrhea Virus

Sera from 147 cows, 16 calves, and 33 heifers were pooled at 10 each, and their RNA extracted using the same kit described previously. In addition, viral RNA was individually extracted from sera of five calves transferred to other farms and 20 newborn calves using the same method.

RT-qPCR for BVDV detection was performed using the same kit and system as described above.

#### 2.4.3. Virus Neutralization Test

The virus neutralization test was performed using sera from 147 cows, 16 calves, and 33 heifers as described previously, to investigate transmission of BVDV in these cattle herds.

## 3. Results

### 3.1. Diagnosis of Eight Calves Based on a Series of Analyses

Upon first sample collection, Ct values positive for BVDV were detected in sera (19.4–33.6) and WBCs (13.0–32.7) from eight calves by RT-qPCR (Table 1). In addition, BVDVs were isolated in sera from four calves (nos. 664, 663, 597, and 711), and WBCs from seven calves (nos. 569, 663, 835, 772, 597, 711, and 761). Moreover, the analysis of AgELISA showed S-N values (0.523–3.744) positive for BVDV in sera of seven calves (nos. 569, 663, 835, 772, 597, 711, and 761). Furthermore, sera from eight calves exhibited relatively low cross-reactivity (<2 to 16) against the BVDV1; however, a wide range of cross-reactivity (<2 to 512) was noted against the BVDV2 in the virus neutralization test.

On the second sample collection after approximately 3 weeks from the first collection, sera and WBCs were collected from seven calves, except from the one that died (no. 761). Ct values positive for BVDV were obtained in sera (27.5–33.7) and WBCs (25.5–37.3) from six calves (nos. 569, 664, 663, 835, 597, and 711) and seven calves using RT-qPCR, respectively. In addition, BVDVs were isolated in serum from one calf (no. 664) and WBCs from three calves (nos. 664, 597, and 711). Moreover, the analysis of AgELISA showed S-N values (0.398–0.516) positive for BVDV in sera from three calves (nos. 664, 835, and 597). Furthermore, sera from seven calves exhibited titers <2 to 64 against the BVDV1 and titers of 8 to 2048 against the BVDV2 in the virus neutralization test.

On the third sample collection after approximately 5–6 weeks from the first collection, sera and WBCs were collected from three calves (nos. 664, 597, and 711). Ct values positive for BVDV were detected in sera (28.3–35.0) of all three calves, and WBCs (30.7–34.3) from two calves (nos. 664 and 597) using RT-qPCR. In addition, no BVDVs were isolated in sera and WBCs of the three calves. Moreover, the analysis of AgELISA using sera from the three calves showed S-N values (0.125–0.244) negative for BVDV. Furthermore, sera from the three calves exhibited titers of 8 to 16 against the BVDV1 and titers of 128 to 1024 against the BVDV2 in the virus neutralization test.

We also performed RT-qPCR and virus isolation using 20% tissue suspensions from the three calves that died (nos. 761, 835, and 711), and one that euthanized (no. 664) (Table 2). RT-qPCR detected Ct values (15.3–37.4) positive for BVDV from all tissue suspensions, other than the liver of one calf (no. 711), prepared from the four calves. BVDVs were isolated from all tissues of the two calves (nos. 761 and 835), except from the liver of one calf (no. 761). In addition, BVDVs were also isolated from the kidney and brain of one calf (no. 711). No BVDVs were isolated from all tissues from the one calf (no. 664).

**Figure 3:**
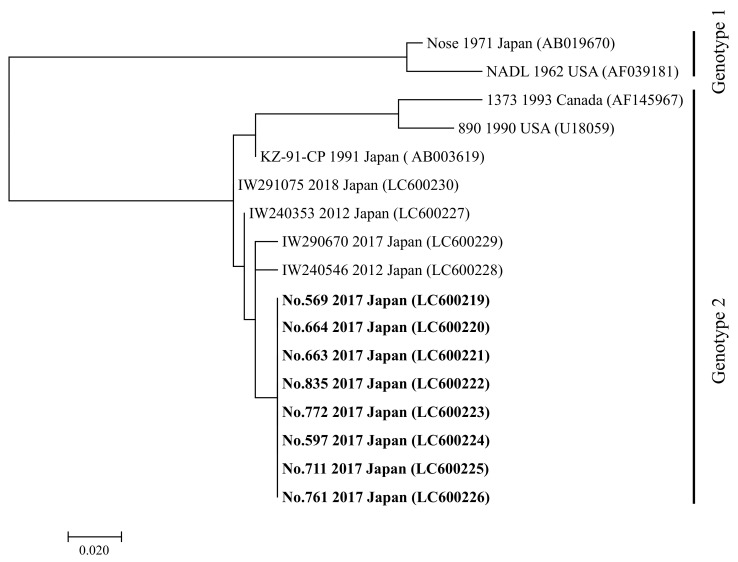
Phylogenetic tree using partial 5′-untranslated region sequences from representative bovine viral diarrhea virus (BVDV) strains. The phylogenetic analysis was performed using nucleotide sequences (product size: 245–248 base pairs) of the 5′-UTR from representative BVDV strains including four BVDV strains (IW240353, IW 240546, IW290670, and IW291075) isolated form cattle with persistent infection in Iwate Prefecture from 2012 to 2018. The tree was constructed using the maximum-likelihood method and 1000 bootstrap replicates on MEGA 7. Collection year, country, and GenBank accession number of strain are shown. BVDV strains isolated from eight calves in this study are shown in bold. BVDV genotypes are shown on the right of the tree. Scale bar represents the number of substitutions per site.

Collectively, these findings showed that eight calves were AI with BVDV. In addition, our analysis revealed that BVDVs detected from these animals were classified into BVDV2 via BVDV genotyping by multiplex RT-qPCR. Moreover, phylogenetic analysis for 5′-UTR genomic sequences classified all BVDVs detected in this study as BVDV2, which was different from high pathogenic BVDV2 strains 890 and 1373 (Figure 3).

### 3.2. BVDV Detection of 16 Calves and 33 Heifers Maintained at Calf Barn, 147 Cows Maintained at Dairy Barn, 5 Calves Transferred to Other Farms, and 20 Newborn Calves

**Figure 4:**
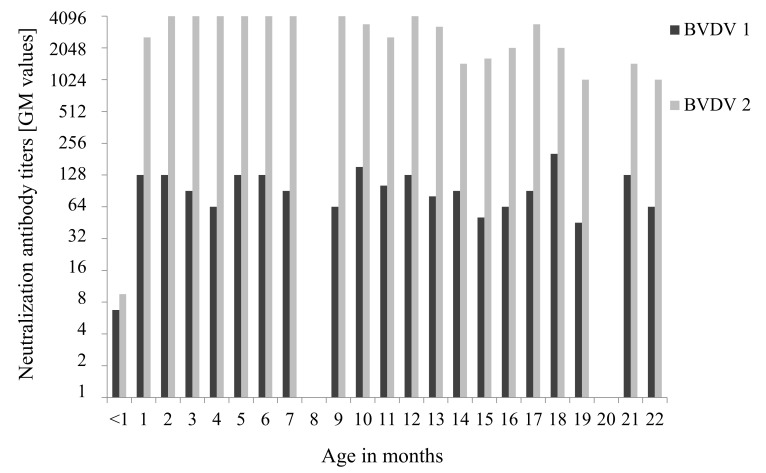
Neutralization antibody titers against bovine viral diarrhea virus 1 (BVDV1) and BVDV2 using sera from 16 calves and 33 heifers maintained at calf barn. Sera were maximally diluted 4096 times. The horizontal axis indicates age in months on day of sample collection. The antibody titers are shown in median values of groups at each age.

**Figure 5:**
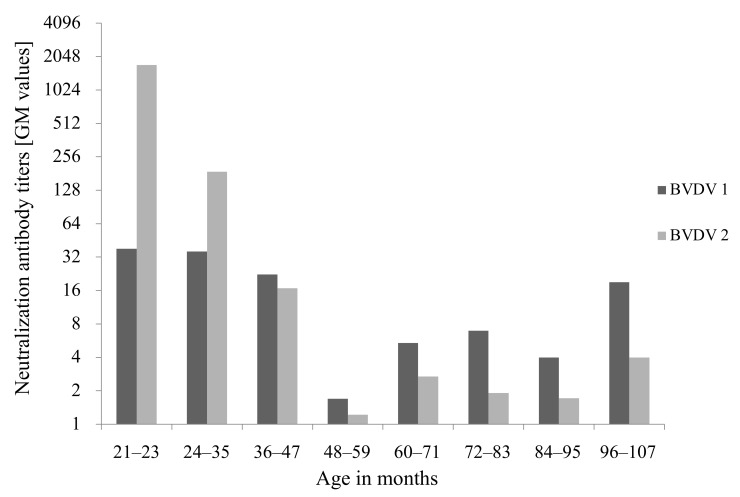
Neutralization antibody titers against bovine viral diarrhea virus 1 (BVDV 1) and BVDV2 using sera from 147 cows maintained at dairy barn. Sera were maximally diluted 4096 times. The horizontal axis indicates age in months on day of sample collection. The antibody titers are shown in median values of groups at each age.

BVDV were not detected in sera of 16 calves and 33 heifers maintained at calf barn, 147 cows maintained at dairy barn, 5 calves transferred to other farms, and 20 newborn calves using RT-qPCR. Neutralization antibody titers against BVDV1 and BVDV2 using sera from 16 calves (<1 to 6 months old) and 33 heifers (7 to 22 months old) were shown by age in months on day (Day 42) of sample collection as follows: <2–64 (geometric mean: 6.7) and <2–32 (geometric mean: 9.5) under 1 month old (*n* = 4), 32–256 (geometric mean: 64–128) and 1024–4096 (geometric mean: 2580.3–4096) at 1–6 months old (*n* = 12), and 16–512 (geometric mean: 45.3–203.2) and 512–4096 (geometric mean: 1024–4096) at 7–22 months old (*n* = 33), respectively (Figure 4). In addition, neutralization antibody titers against BVDV1 and BVDV2 using sera from 147 cows were exhibited at intervals of 12 months based on age in months on day (Day 95) of sample collection as follows:16–128 (geometric mean: 38.1) and 1024–4096 (geometric mean: 1722.2) at 21–23 months old (*n* = 4), <2–1024 (geometric mean: 35.9) and <2–4096 (geometric mean: 188.1) at 24–35 months old (*n* = 36), <2–4096 (geometric mean: 22.3) and <2–4096 (geometric mean: 16.8) at 36–47 months old (*n* = 44), and <2–512 (geometric mean:1.7–19.0) and <2–64 (geometric mean:1.2–4.0) at 48–107 months old (*n* = 63) (Figure 5).

## 4. Discussion

This study demonstrated that BVDV AI cattle might be a source of transmission to the herds, resulting in a continuous outbreak. Although previous studies have reported virus transmission from AI animals to other animals in controlled challenge studies [24,37], to our knowledge, this is the first report of virus transmission from AI animals to other animals in a field outbreak. 

On the first sample collection, Ct values of RT-qPCR for sera and WBCs from five calves (nos. 569, 664, 663, 597, and 711) were 19.4–22.5 and 13.0–21.0, respectively, which were similar to those of sera and WBCs from PI calves [38]. In addition, S-N values measured using sera from four calves (nos. 569, 663, 597, and 711) could not be distinguished from those obtained using sera from PI calves [38]. On the second sample collection, approximately 3 weeks from the first collection, Ct values of RT-qPCR using sera and WBCs from three calves (nos. 569, 663, and 772) showed a clear increase. In addition, virus isolation and S-N values of AgELISA using sera from the three showed no isolation and a decrease, respectively. These data showed that the three calves were not PI animals, but could not diagnose whether the three remaining calves (nos. 664, 597, and 711) were PI or AI animals. On the third sample collection, approximately three additional weeks from the second collection, Ct values of RT-qPCR using sera and WBCs from three calves showed a clear increase as compared to those in the first or second sample collection. In addition, no BVDVs were isolated from the samples of the three calves. Moreover, the titers against BVDV2 measured by the virus neutralization test showed a clear increase as compared to those in the first or second sample collection. These data also exhibited the three remaining calves were not PI animals. Collectively, we concluded that the eight calves with clinical symptoms were AI with BVDV2 based on a series of analyses including RT-qPCR, virus isolation, AgELISA, and virus neutralization test using their sera and/or WBCs collected from one to three sample collection throughout a period of 6 weeks. 

The OIE manual recommends that infected animals should be re-tested virologically and serologically using blood samples collected after an interval of three additional weeks from first sample collection to diagnose BVDV PI [39]. In contrast, we took up to 6 weeks and three sample collections to diagnose that the eight calves were AI with BVDV2 in this study. Therefore, our results suggest that investigation with various tests throughout multiple sample collection is important to clearly distinguish between BVDV AI and PI. 

“How long will AI cattle excrete BVDVs?” A previous study showed that BVDVs were detected in sera, blood, WBCs, and nasal secretions from AI postnatal animals (cattle and sheep) 4–10 days post-infection [20]. In contrast, other studies showed that BVDVs were detected in blood and WBCs from AI animals after 85 days and 95 days post-infection, respectively [24,37]. In this study, BVDV2 was isolated from the kidney and brain of the calf (no. 711) that died on 34 days after the first sample collection. In addition, the BVDV2 gene was detected in several tissues of the calf (no. 664), which euthanized on Day 102. Particularly, the former showed that BVDV2 had long term infectivity within the body of the calf. Therefore, the data presented herein suggest that AI calves excreted the virus for a prolonged duration, which would be the source of transmission to other calves. 

A previous study reported an outbreak of severe diarrhea by a highly virulent BVDV strain, CD87, in the state of New York [40]. During this outbreak, infected cattle showed severe clinical signs, such as high fever, diarrhea, rapid decrease of milk production, morbidity of 50%, and mortality of 20%. Thereafter, several highly virulent BVDV strains with severe symptoms have been reported [27,41,42,43]. In addition, case reports of AI with hemorrhagic lesions, thrombocytopenia, and high mortality have been continuously reported [40,44,45]. The severity of symptoms caused by BVDV might be closely related to virus virulence and their interaction with other pathogens [46,47,48,49]. The case report presented in this study was a sporadic occurrence of BVDV2 AI, because 11 calves (three other calves in addition to the above eight calves) of 57 cattle maintained at calf barn had diarrhea and/or respiratory disorders. In addition, the infection rate of BVDV in the 57 cattle was 100%, because of seroconversion and/or an increase in neutralization antibody titers against BVDV2 in all calves; however, the mortality was 5.3% (3/57). Moreover, phylogenetic analysis showed BVDVs isolated in this study were classified into clusters different from the cluster into which high virulent BVDV strains belonged. These findings suggest that these BVDVs might be low pathogenic BVDV2 strains, and not highly pathogenic ones. Although bovine rotavirus A has been detected in feces of some calves, other pathogens have been not been identified in the samples used in this study.

Although it is known that each neutralizing antibody against BVDV1 and BVDV2 reciprocally cross-reacted against the two genotypes, it can consider be either genotype-specific reaction when there was a difference of higher than 4-fold between the titers against each genotype [50]. Based on the criteria, our data revealed that the neutralizing antibody titers from the cattle (1–35 months old) maintained in this farm were specific against BVDV2 not BVDV1. In contrast, the delivered cows (36–107 months old) maintained in this farm showed neutralizing antibody titers (geometric mean: 1.7–22.3 and geometric mean: 1.2–16.8) against BVDV1 and BVDV2, respectively. A previous study reported that the neutralizing antibody titers with ≤64 against BVDV could not effectively protect animals from BVDV infection [51,52]. Therefore, our data also suggested that the titers from the delivered cows might be not enough to prevent BVDV infection.

Our analysis showed that both neonatal calves (under 1 month old) and delivered cows not less than 36 months old had low neutralizing antibody titers against BVDV2 (geometric mean: 1.2–16.8). In addition, the eight AI calves were 11–67 days of age on the first sample collection, five of which were less than 1 month old. It is important to give an enough passive immunity via colostrum due to protect newborn calves from infection of pathogen. However, newborn calves were fed pooled colostrum which had been collected and stocked from the delivered cows in this farm. Therefore, these facts suggest that these calves were unable to receive enough passive immunity against BVDV2 via colostrum with low neutralizing antibody titers and were consequently infected with these viruses. Generally, calves under 1 month of age could not activate sufficient self-immunity, which might have prolonged their BVDV2 infections [53].

One of the pitfalls of this field study is the possibility of reinfection as these eight calves were housed in the same barn without any isolation from each other. BVDV AI were continuously occurring in the calf barn, and the viruses shed from the AI animals were always present there. Thus, there was the possibility that many calves may have been exposed to BVDVs. In general, BVDV-infected cattle usually acquire strong immunity that should prevent the reinfection. However, in this case, a rapid antibody elevation did not occur in some calves, meaning that reinfection could have occurred prior to the acquirement of BVDV immunity. Future studies need to be done with higher biosecurity to prevent any possibility of reinfection between calves to investigate true nature of the virus shedding and spread from BVDV AI animals.

A previous study suggested the possibility of transmission from AI animals to other animals, based on a case report in which seroconversion occurred without PI animals [30]. In this study, we examined the existence of PI animals using 229 cattle (24 calves, 33 heifers, 147 cows, 5 calves transferred to other farms, and 20 newborn calves) on this farm over a period of four months; however, we found eight calves with BVDV2 AI and not PI. In addition, genetic analysis using the 5′-UTR sequences of BVDV from the eight AI calves showed that the origin of BVDV2 among them was identical. Moreover, both cattle herds were not in contact, because the delivery barn and the dairy barn were completely separated on the same farm. Furthermore, newborn calves are moved to the calf barn within 1 day. Therefore, there was no transmission of the virus from the delivery room to the dairy barn, even if a PI calf was born. In contrast, a portion of the calves were transferred to other farms for breeding. The fact suggested that the cattle maintained on those farms might be infected with BVDV2 during early pregnancy. Moreover, the fetus might also be infected with BVDV2. Collectively, this suggests the possibility that the virus may have been brought into the calf barn by the PI foetus, a so-called “Trojan horse”, as an invasion route of BVDV2 onto this farm.

The possibility of transmission of BVDV from AI animals to other susceptible animals still remains controversial as reported in the previous studies [29,30,54,55,56]. In addition, efficient transmission of BVDV requires exposure to higher viral loads and for a longer period of time. In this field case, it suggests that the existence of a large number of newborn calves susceptible to BVDV2 played a major role in the maintenance and continuation of BVDV2 in the calf barn. To our knowledge, this study strongly suggets the possibility of BVDV transmission from AI animals to other susceptible animals. 

In conclusion, the diagnosis of eight calves with diarrhea and/or respiratory disorders were AI with a low virulent BVDV2 strain, not a highly virulent one, by repeating multiple tests throughout 6 weeks. Our results suggest that AI might be transmitted to other calves, and therefore might continue to occur among multiple naïve calves for a long time without the existence of PI animals. This study is a valuable report that demonstrated the clinical characteristics of BVDV2 AI in the field. The data presented herein provides important information to broaden the understanding of different disease types caused by BVDV. Further accumulation of information via multiple analyses for a field case of BVD outbreak would contribute to diagnose rapidly and accurately BVDV AI and PI in future.

## Figures and Tables

**Table 1:** Summary of Ct values by real-time RT-PCR (RT-qPCR), results of virus isolation, S-N values of antigen detective enzyme-linked immunosorbent assay (AgELISA), neutralization antibody titers against BVDV1 and BVDV2, and BVDV genotype determined by multiplex real-time RT-PCR (multiplex RT-qPCR) in diagnosis of eight calves with clinical symptoms.

Calf No	Age at Initial BVDV Detection (Days Old)	Timing of Sample Collection	Sample Collection Date	Days of Sample Collection ^a^	Ct Value by RT-qPCR ^b^		Virus isolation ^b^		AgELISA (S-N Values)	Neutralization Antibody Titers		Genotype	Clinical Symptoms
					Sera	WBCs	Sera	WBCs		BVDV1	BVDV2		
569	21	1st	2017/3/31	0	20.7	19.3	−	+	3.744	<2	16	BVDV2	diarrhea
		2nd	2017/4/21	21	32.1	28.4	−	−	0.025	2	512	BVDV2	diarrhea
664	38	1st	2017/3/31	0	22.5	21.0	+	−	0.125	<2	<2	BVDV2	diarrhea and respiratory disorder
		2nd	2017/4/21	21	27.5	25.5	+	+	0.479	<2	8	BVDV2	
		3rd	2017/5/8	38	34.4	34.3	−	−	0.169	16	1024	BVDV2	
		4th	2017/7/11	102	Undetermined	Undetermined	−	−	0.010	256	>4096	BVDV2	**poor growth (necropsy)**
663	11	1st	2017/4/13	13	21.3	18.7	+	+	3.727	<2	<2	BVDV2	respiratory disorder
		2nd	2017/5/8	38	32.1	35.0	−	−	0.142	4	64	BVDV2	respiratory disorder
835	67	1st	2017/5/8	38	33.6	32.7	−	+	0.523	16	64	BVDV2	respiratory disorder
		2nd	2017/5/17	47	33.7	28.5	−	−	0.516	8	64	BVDV2	**died (necropsy)**
772	22	1st	2017/5/12	42	29.6	29.5	−	+	1.391	<2	2	BVDV2	mild respiratory disorder
		2nd	2017/6/2	63	Undetermined	37.3	−	−	−0.006	64	2048	BVDV2	
597	13	1st	2017/5/12	42	19.9	13.0	+	+	3.286	<2	<2	BVDV2	mild respiratory disorder
		2nd	2017/6/2	63	30.0	26.8	−	+	0.398	<2	16	BVDV2	
		3rd	2017/6/23	84	35.0	30.7	−	−	0.125	16	256	BVDV2	
711	17	1st	2017/5/12	42	19.4	19.3	+	+	3.672	<2	2	BVDV2	bloody stool
		2nd	2017/6/2	63	33.0	27.6	−	+	0.013	2	128	BVDV2	bloody stool, respiratory disorder
		3rd	2017/6/15	76	28.3	Undetermined	−	−	0.244	8	128	BVDV2	**died (necropsy)**
761	37	1st	2017/6/15	76	25.5	Undetermined	−	+	1.568	8	512	BVDV2	bloody stool, respiratory disorder, **died (necropsy)**

^a^ The day collected samples from the two calves (nos. 569 and 664) was defined as Day 0. ^b^ RT-qPCR and virus isolation were performed using sera and white blood cells (WBCs) from the eight calves. +: positive, −: negative for virus isolation. Bold represents died and euthanized individuals.

**Table 2:** Summary of Ct values by real-time RT-PCR (RT-qPCR) and results of virus isolation using 20% tissue suspensions of the four necropsied calves

Calf No	Age at Initial BVDV Detection (Days Old)	Timing of Sample Collection	Day after Initial Detection of Each Calves	Ct values by RT-qPCR								Results of virus isolation ^a^							
				Superficial Cervical Lymph Node	Subiliac Lymph Node	Kidney	Spleen	Liver	Lung	Heart	Brain	Superficial Cervical Lymph Node	Subiliac Lymph Node	Kidney	Spleen	Liver	Lung	Heart	Brain
664	140	4th	102	23.4	23.2	29.5	34.6	37.4	33.5	29.2	30.3	−	−	−	−	−	−	−	−
835	76	2nd	9	16.4	15.7	20.0	22.3	29.0	23.5	22.8	24.1	+	+	+	+	+	+	+	+
711	51	3rd	34	17.4	15.3	17.1	20.6	Undetermined	26.5	22.0	24.4	−	−	+	−	−	−	−	+
761	37	1st	0	19.7	20.0	18.7	22.6	25.0	21.0	20.8	26.2	+	+	+	+	−	+	+	+

^a^ +: positive, −: negative for virus isolation.

## Data Availability

No new data were created or analyzed in this study. Data sharing is not applicable to this article.
